# Bacterial Infection in Deep Paraspinal Muscles in a Parturient Following Epidural Analgesia: A Case Report and Literature Review: A CARE-Compliant Article

**DOI:** 10.1097/MD.0000000000002149

**Published:** 2015-12-18

**Authors:** Xuhong Xue, Jiefu Song, Qingyuan Liang, Jibin Qin

**Affiliations:** From the Department of Orthopedics, The Second Hospital, Shanxi Medical University (XXH); Department of Orthopedics, Shanxi Provincial People's Hospital, Shanxi Medical University (SJF); Department of Orthopedics, Shanxi Provincial People's Hospital, Shanxi Medical University (LQY); and Department of Orthopedics, Shanxi Provincial People's Hospital, Shanxi Medical University, Taiyuan, Shanxi, P.R. China (QJB).

## Abstract

Bacterial infection related to epidural catheterizations could occur. In general, the incidence of postoperative infection at the insertion site is very low. Paucity literatures are reported for paraspinal muscle infection after epidural analgesia in parturient. We report a case of paraspinal muscle infection shortly after epidural analgesia in a parturient, who was subjected to because of threatened preterm labor. Epidural morphine was administered for 2 days for childbirth pain control. She began to have constant low-back pain and fever on postpartum Day 2. Magnetic resonance image revealed a broad area of subcutaneous edema with a continuum along the catheter trajectory deep to the paraspinal muscles. A catheter-related bacterial infection was suspected. The surgical debridement and drainage was required combined with intravenous antibiotics on postpartum Day 3. She was soon cured uncomplicatedly.

Epidural analgesia is effective to control labor pain and, in general, it is safe. However, the sequelae of complicated infection may be underestimated. A literature search yielded 7 other cases of catheter-related epidural abscess or soft tissue infection. Vigilance for these infections, especially in postpartum patients with backache, is needed. Moreover, early detection and proper treatment of infectious signs at postanesthetic visit are very important.

## INTRODUCTION

Epidural analgesia is effective in treating the pain experienced by parturients in labor, but infection may occur after the procedure.^1^ Infection related to epidural catheterizations could occur superficially, deep, or in the epidural spaces.^2,3^ In general, the incidence of postoperative infection at the insertion site is very low. Nine cases of epidural abscesses were reported in a series of 17,372 epidural catheters in a prospective study. The incidence of was 0.052%.^4^ The rates of deep muscle and epidural infections were reported to be less than 0.001%.^5^ Though the reported infection rates are quite low,^4^ the sequelae could be serious and life threatening without early diagnosis and treatment.

Back pain is an early sign of epidural analgesia-related infection, but may be ignored post partum when generalized backache is common.^6,7^ We herein report a pregnant woman who received epidural analgesia for labor pain. She received epidural morphine for labor pain management. A broad area of subcutaneous infection found concentrically to the epidural injection site associated with paraspinal muscle fasciitis was diagnosed on postpartum Day 2. A disastrous complication of dissemination from deep muscles to serious neuraxial infection is possibly avoided.

## ETHICS AND CONSENT

The study was approved by the Ethical Committee for Shanxi Provincial People's Hospital, Shanxi Medical University. Written informed consent was obtained from the patient for publication of this case report and any accompanying images. A copy of the written consent is available for review by the editor of this journal.

## CASE REPORT

A 40-year-old pluripara at 35 weeks’ gestation was admitted for threatened preterm labor. She was free from systemic diseases, such as hypertension, diabetes, or immunological problems, and had no history of spinal abnormality or chronic back pain. Because of premature rupture of membrane and her blood examination on admission, which showed leukocytosis with white cell count of 20.73 × 10^9^/L, 2 g of intravenous ampicillin was prophylactically given once. After admission, regular uterine contraction come, she requested painless labor.

After detailed explanation, epidural catheterization was performed by an anesthesiologist. Following disinfection and draping an 18-gauge Tuohy needle was inserted through L3-L4 interspinal space under local anesthesia with 1% lidocaine. After confirmation of right epidural entry, an epidural catheter was introduced until an 8-cm segment of the catheter was in the epidural space. A bacterial filter was placed at the distal end of the catheter before connection to the infusion pump. No blood or cerebrospinal fluid was aspirated from the catheter and no oozing of fluid was noted at the puncture site. Continuous epidural infusion with a solution of fentanyl 2 μg/mL and ropivacaine 0.1% in normal saline was started immediately after epidural catheterization. Her pain score decreased from 7 to 8 before injection to 2 to 3 30 min after injection, as evaluated by a 0 to 10 verbal rating scale.

Five hours later, health baby weighing 2650 g was delivered uneventfully. Laceration of the perineum was found in parturient who was treated with perineal laceration suturing. The epidural catheter for labor pain was not removed; postoperative pain control was started immediately after delivery with a bolus of 2 mg morphine in 10 mL normal saline injected in the epidural space followed by 2 mg of epidural morphine given every 12 h by our pain service staff.

The patient was visited twice daily and she complained of only low grade pain (verbal rating scale 2–3). On postpartum Day 2 (∼48 h after epidural insertion), the epidural catheter was removed. A 1.0 cm by 1.5 cm erythematous, swollen papule with little purulent discharge was noted at the epidural injection site. Besides, she complained of serious low-back pain but the bilateral leg sensation and muscle power were unmolested. Physicians noted a small amount of discharge from the insertion site, and the body temperature was elevated to 39°C.

**FIGURE 1:**
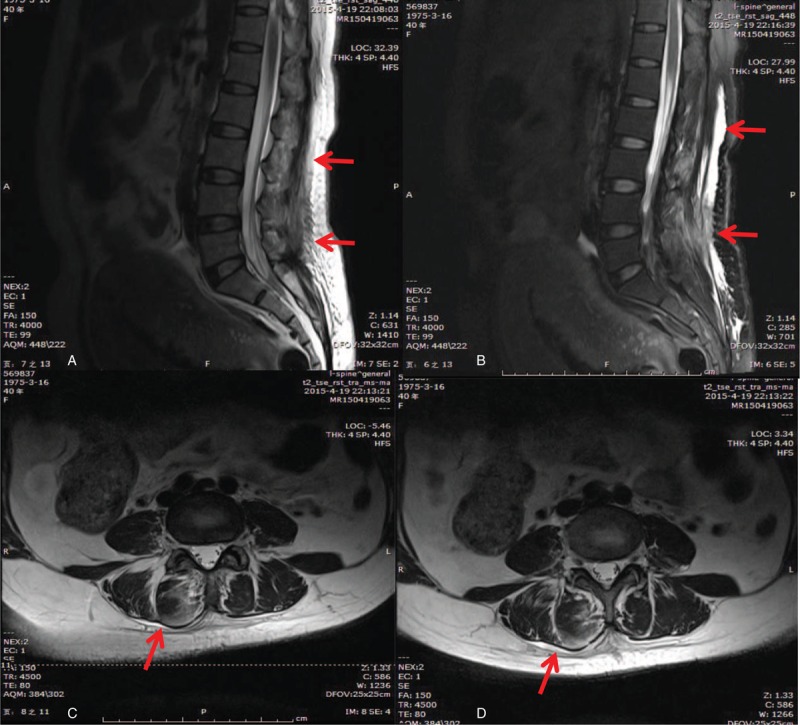
T2-weighted magnetic resonance image scanning without contrast medium on the postpartum Day 2 showed paraspinal muscles infection related to epidural injection. (A) Median sagittal view: the high-intense signal spreads from the subcutaneous tissues to the deep tissues between the L1-L5 interspinal process, indicating a wide and deep muscle involvement. No signal was shown at the epidural space. (B) Median sagittal fat-suppression sequence: a large edematous inflammation/(signal) spreading over subcutaneous tissues (encircled by red arrows). (C) An axial-view T2 image: a large inflammation extending from the superficial subcutaneous tissues to bilateral sides of deep paraspinal muscles (more at the right side) along the epidural injection tract in the L2/3 level. (D) An axial-view T2 image: a large inflammation extending from the superficial subcutaneous tissues to bilateral sides of deep paraspinal muscles (more at the right side) in the L3/4 level.

Under suspicion of epidural or paraspinal muscles infection, a magnetic resonance imaging (MRI) was immediately arranged and empiric antibiotic with 2 g of intravenous cefoxitin every 12 h was started. The images suggested a deep lumbar infection with evident findings over a broad area from subcutaneous tissues beneath the epidural injection site, extrapolating to bilateral deep paraspinal muscles (Fig. 1). It extended from the L2/3 to L4/5 levels. The blood test indicated that the white cell count was 18.87 × 10^9^/L with 88.4% neutrophils and the C-reactive protein was 110 mg/dL. The diagnosis was a bilateral paravertebral soft tissue infection without evidence of an epidural abscess or osteomyelitis. An orthopedic opinion was sought, and the patient underwent debridement and drainage on postpartum Day 3. During the operation, ∼20 mL of turbid liquid was drained. The thoracolumbar fascia was invaded and paravertebral soft tissue edema was found. A pus swab taken during the surgery grew oxacillin-sensitive *Staphylococcus aureus* with sensitivity to cefotiam and levofloxacin. The antibiotics were then changed to cefotiam and levofloxacin. Low-back pain improved on postoperation day 1. The erythematous papule decreased in size and was fading. The papule and clinical symptoms were resolved on postoperation Day 5 and laboratory data turned for normal on postoperation Day 10 completely. Patient was discharged on postoperation Day 16. No fever was noted throughout the course.

## DISCUSSION

A clinically significant soft-tissue infection, not involving the spinal epidural space, is rare in the parturient who has had an epidural catheter sited. A MEDLINE search of the National Library of Medicine database yielded 5 additional cases of catheter-related epidural abscess and 2 cases of catheter-related soft tissue infection in the parturient.^3,5,8–12^Tables 1 and 2 summarize the clinical features of all 7 patients with epidural abscess or soft tissue infection. We report an epidural analgesia-related paraspinal deep muscle infection in a parturient under spinal anesthesia. It might thus have prevented a serious neurological deficits complication if left undiagnosed.

**TABLE 1:**
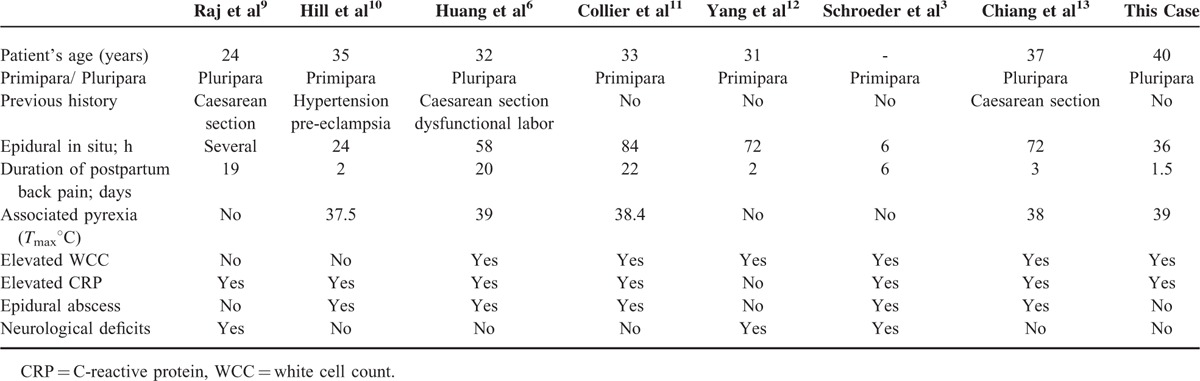
Clinical Presentation of These Cases of Significant Soft-Tissue Infections or Epidural Abscess and Associated With a Labor Epidural

**TABLE 2:**
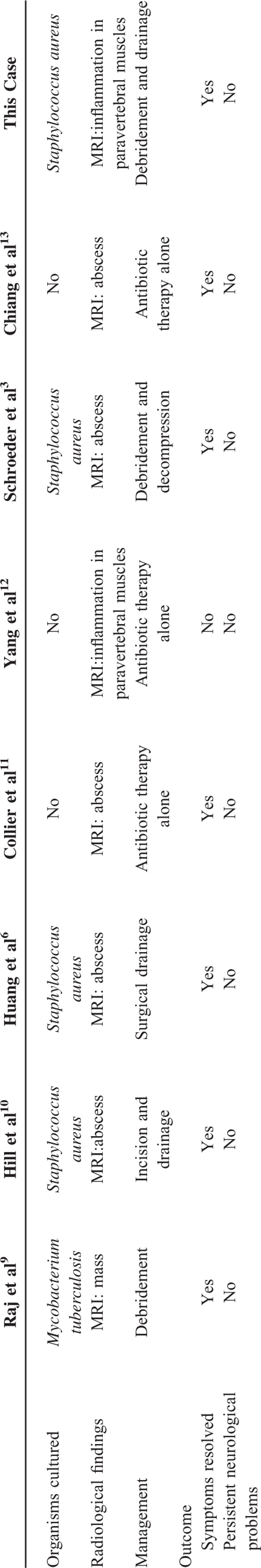
Characteristics, Treatment, and Clinical Outcomes for Patients Who Developed Spinal Epidural Abscess After the Use of Temporary Epidural Catheters

In our and other 7 cases, postpartum back pain was a presenting symptom. It should be considered the possibility of a spinal epidural abscess with the progression of this symptom.^10,13^ Furthermore, the serious complications of epidural catheterization, such as neurological deficit may be presented if not appropriately diagnosed and managed. Heusner ^7^ described the clinical progression of spontaneous epidural abscess phases, which proceeded over various time intervals. However, not all spinal epidural abscesses follow exactly this course.

Although deep muscle infections after labor were sporadically reported, they may be underestimated. Any types of infections related to epidural techniques are potentially detrimental as there is risk of progression to epidural abscess.^14^ It was reported that many pre-existing pathologies potentially liable patients to epidural-related infection, such as diabetes, malnutrition, or immune-compromised condition in previous studies.^15,16^ Phillips et al assumed that a contaminated intrusion and spreading of bacteria from the epidural catheter are possible mechanisms.^17^ Moreover, peripartum may be highly liable to be infected because of particular conditions, such as preterm rupture of membrane, episiotomy, or surgery.

In the present report, the patient might have sustained the epidural injection in the delivery room where the environment is less aseptic than the operation room. Presumably, skin-born microbe may be the cause of the wide spreading subcutaneous infection in the light of these vulnerable practices, and reading of MRI images however genuine is impossible to confirm. No matter how infectious complications are unpredictable and unavoidable, they can be substantially reduced by strictly preventive maneuvers, such as absolute skin disinfection, aseptic injection technique, careful implantation care, and standardized health care protocols. In this case, the MRI images showed an edematous change along the injection trajectory to deep paraspinal muscle groups suggesting an infectious progression, which was consistent with the development of symptoms in the patient, that is, back pain, then continuous fever. A study of postoperative epidural patients indicated that positive culture of bacteria in the subcutaneous catheter was as high as 10%, which suggested that bacterial migration along the epidural catheter tract from skin insertion site to catheter tip in the epidural space was the most common route of epidural colonization.^18^ When bacteria invade deeply enough to neuraxial tissues close to epidural or intrathecal spaces, some complications, including meningitis, paraplegia, and even death, may occur. In this case, we diagnosed the infection early enough before it had become severe and serious and a catastrophic sequela was prevented.

The causative organism is nearly always *S. aureus*, which frequently comes from the skin. This is consistent with our study. It was confirmed by wound culture. A recent report by Masanovu et al suggested that hyperhidrosis should be considered as a potential risk for epidural abscess.^19^ Back pain, the first sign we found in this case, occurred on postpartum Day 2. Other infectious signs, such as skin discharge and fever, were delayingly found or not obvious, probably because of antibiotic use for surgical purpose. More importantly, several available reports indicated that epidural-related deep infections in postpartum women were not diagnosed until 19 to 22 days after delivery ^5,8,10^ at which time most of the patients had been discharged from hospital.

Several measures including early diagnosis, surgical exploration and drainage, and combined with appropriate antibiotics should be carried for the patients with epidural abscess or paraspinal muscle infections. In our case and these 7 others, surgical decompression was not required in 5 patients without significant neurological deficits. All of them have recovered following antibiotic treatment or drainage. However, the risk of neurological impairment could be existed. As the outcome is closely related to the length of time the abscess has been present.^20,21^ Thus, the surgical intervention should be indicated in the patient complicating significant neurological deficits or progressing spinal abscess as soon as possible. Of course, conservative treatment with antibiotics has been used with good outcome in the absence of neurological signs.^11,12^

Because pregnancy-induced low-back pain and injection induced pain can be combinedly present in parturients, any newly coming back pain should be distinguished. If a female complained of low-back pain following epidural treatment, meticulous investigations to differentiate neuraxial infection, epidural hematoma, or neurotrauma have to be started immediately. In addition to diagnosing from clinical symptoms, physical examinations, and laboratory studies, MRI can efficiently help to reach a fast and reliable diagnosis and to make a decision for a conservative or surgical treatment.^22^ In some reports, computed tomography scan without myelography provided little diagnostic information and not considered to be the method of choice.^23^ We arranged MRI as soon as the patient complained of back pain and timely prevented an insidious development of neuraxial abscess formation.

## CONCLUSIONS

In a word, a case of epidural catheter-related infection was reported in a parturient. It was suggested that not all infectious complications involve the spinal epidural space. Anesthetists should be aware of this possibility, so as to avoid the serious subsequent. Moreover, early detection and proper treatment of infectious signs at postanesthetic visit are very important. To reduce catastrophic consequence, all fail-safe steps at preinjection evaluation, standardized interventional procedures, and postinjection care should be always considered significant.
